# Knock-down of microRNA miR-556-5p increases cisplatin-sensitivity in non-small cell lung cancer (NSCLC) via activating NLR family pyrin domain containing 3 (NLRP3)-mediated pyroptotic cell death

**DOI:** 10.1080/21655979.2021.1971502

**Published:** 2021-09-07

**Authors:** Feng Shi, Luquan Zhang, Xing Liu, Yue Wang

**Affiliations:** a Department of Medical Oncology, Harbin Medical University Cancer Hospital, Harbin, China; b Department of Thoracic Surgery, Harbin Medical University Cancer Hospital, Harbin, China; c Department of Orthopedics, The First Affiliated Hospital of Harbin Medical University, Harbin, China; d Department of Pharmacology and Toxicology, Wright State University, Dayton, OH, USA

**Keywords:** miRNAs, non-small cell lung cancer, NLRP3, miR-556-5p, cell pyroptosis

## Abstract

MicroRNAs (miRNAs) are small non-coding RNAs that are closely associated with cancer progression and drug resistance, however, up until now, the involvement of miR-556-5p in regulating cisplatin-sensitivity in non-small cell lung cancer (NSCLC) has not been studied. In the present study, we found that miR-556-5p was significantly upregulated in the cisplatin-resistant NSCLC (CR-NSCLC) patients’ tissues and cells, instead of the corresponding cisplatin-sensitive NSCLC (CS-NSCLC) tissues and cells. Further experiments validated that knock-down of miR-556-5p suppressed cell viability and tumorigenesis, and induced cell apoptosis in the cisplatin-treated CR-NSCLC cells, and conversely, upregulation of miR-556-5p increased cisplatin-resistance in CS-NSCLC cells. Interestingly, miR-556-5p ablation triggered pyroptotic cell death in cisplatin-treated CR-NSCLC cells via upregulating NLRP3, and the promoting effects of miR-556-5p silence on cisplatin-sensitivity in CR-NSCLC cells were abrogated by both cell pyroptosis inhibitor NSA and NLRP3 downregulation. Taken together, this study firstly evidenced that induction of NLRP3-mediated cell pyroptosis by miR-556-5p downregulation was effective to increase cisplatin-sensitivity in NSCLC, which provided new therapy strategies to overcome chemo-resistance for NSCLC patients in clinic.

## Background

Non-small cell lung cancer (NSCLC) is the most common subtype of lung-associated malignancy worldwide [1,2], which brings huge health burden for human beings. The traditional therapy treatments for NSCLC include surgical resection, radiotherapy, chemotherapy, immunotherapy, and so on [3,4]. However, the therapeutic efficacy of the above therapies is seriously limited by their inevitable shortcomings [3,4], and although great advances have been reached in the development of novel treatment strategies, there is still no effective therapies for the treatment of metastatic NSCLC. As for the most commonly used chemotherapies, the chemical drugs were effective to kill NSCLC cells at the beginning. Unfortunately, as the results of the generation of drug-resistance under long-term cisplatin treatment, the initial chemo-sensitive NSCLC cells become resistant to those drugs, which makes them ineffective to kill cisplatin-resistant NSCLC cells [5–8]. The underlying mechanisms are very complicated, and previous publications hint that cancer stem cells (CSCs) properties [7,9,10] and cell autophagy [5,11,12] may be crucial for this process. Among all the chemical drug, cisplatin (DDP) is commonly used for NSCLC treatment, and cisplatin-resistance is also a unbridgeable obstacle which makes this drug ineffective [13–15], and investigations in this issue become urgent and necessary.

Currently, researchers focus on uncovering the underlying mechanisms by which NSCLC cells generate resistance to cisplatin, and a large variety of cancer-related genes are identified to regulate cisplatin-resistance [14,16,17]. Those genes, including oncogenes and tumor suppressors, play important roles in regulating cancer progression and drug resistance in NSCLC [14,16,17]. Especially, various NSCLC-related microRNAs (miRNAs) with post-transcriptional activities are recently identified to modulate cisplatin-resistance [18–22]. For example, Hong et al. verify that let-7 miRNA regulate cell stemness and immune evasion to influence cisplatin-resistance in NSCLC [16], and Wu et al. report the involvement of miR-193a in regulating cisplatin-resistance [15]. Among all the miRNAs, the role of miR-556-5p in regulating cancer progression varies according to cancer types, which acts as an oncogene to promote cancer development in prostate cancer [23], while Zhou et al. [24] and Zhang et al. [25] report that upregulation of miR-556-5p suppresses triple-negative breast cancer and meningioma progression. Nevertheless, up until now, the involvement of miR-556-5p in regulating drug resistance in NSCLC has not been studied.

According to the published literatures, various types of cell death, including cell apoptosis [26–28], necroptosis [29,30], ferroptosis [31,32], and pyroptosis [33–35], involve in regulating cisplatin-resistance in cancers. Especially, cell apoptosis [26–28] and pyroptosis [33–35] are two types of programmed cell death that participate in the regulation of cisplatin-resistance in NSCLC, and researchers notice that there exist interplays between apoptotic and pyroptotic cell death [36,37]. In addition, the apoptotic [38,39] and pyroptotic [40,41] processes can be controlled by miRNAs, and researchers find that miR-556-5p affects cell apoptosis in meningioma [25] and cholangiocarcinoma [42], and Yu et al. evidence that upregulation of miR-556-5p inactivates NLRP3-mediated cell pyroptosis to ameliorate ovalbumin (OVA)-induced allergic rhinitis (AR) [43].

Collectively, the aims of this study were to investigate the role and underlying mechanisms of the miR-556-3p/NLRP3 axis mediated cell pyroptosis in regulating cisplatin-sensitivity in NSCLC, and this work will provide potential strategies to improve cisplatin-sensitivity in NSCLC.

## Materials and methods

### Collection and analysis of the clinical specimens

The Ethics Committee Affiliated to Harbin Medical University Cancer Hospital approved our clinical experiments, and all the participants signed the informed consent forms. The total cohort of NSCLC patients (N = 47) were recruited in Harbin Medical University Cancer Hospital during 2015–2019, and their cancer tissues were collected by surgical resection, which were immediately stored at −80 °C conditions for further utilization. According to their therapy history, those specimens were divided into two groups with or without cisplatin treatment, and the patients (N = 27) with cisplatin treatment history were considered as cisplatin-resistant, whereas patients (N = 20) without chemotherapy history were deemed as cisplatin-sensitive. The clinicopathological characteristics of those patients were listed in Table 1.

**Table 1.** The correlations of miR-556-5p levels with the clinicopathological characteristics of NSCLC patients

Parameters	Cases	miR-556-5p		*P* value
		High	Low	
Age				0.232
≥50	32	16	16	
<50	15	5	10	
Gender				0.162
Male	29	17	12	
Female	18	4	14	
TNM stage				**0.023**
I/II	23	10	13	
III/IV	24	11	13	
Smoking				0.411
No	31	16	17	
Yes	16	5	11	

### Cell culture and treatment

The parental cisplatin-sensitive NSCLC (CS-NSCLC) cells (A549 and H1299) were purchased from The Chinese Academy of Sciences Cell Bank (Shanghai, China), and the cells were cultured in the DMEM medium (Gibco, CA, USA) supplemented with 10% fetal bovine serum (FBS, Gibco, CA, USA), 1% penicillin and streptomycin (Sigma-Aldrich, St. Louis, MO, USA). The cells were placed in an incubator with 5% CO_2_ humidified air at 37°C, and the CS-NSCLC cells were subjected to long-term low-dose cisplatin exposure (0.5–5 μg/ml, 80 days) to establish cisplatin-resistant NSCLC (CR-NSCLC) cells (A549/DDP, H1299/DDP) following the experimental procedures provided by the previous work [44], and the CR-NSCLC cells were maintained in the complete medium containing 1 μg/ml cisplatin, which were subsequently subjected to high-dose cisplatin (25 μg/ml) treatment and 1 mM of pyroptosis inhibitor Necrosulfonamide (NSA, HY-100573, MedChem Express) for 0 h, 6 h, 12 h, 18 h, 24 h and 48 h.

### Vectors transfection

The miRNA-NC, miR-556-5p mimic and inhibitor, and NLRP3 ablation vectors were designed as previously reported [42,43], and were constructed by a commercial third-party company (GenePharma, Shanghai, China), which were then transfected into the NSCLC cells by using the Lipofectamine 2000 reagent (Invitrogen, CA, USA) in keeping with the producer’s instructing procedures. The transfection efficiency of the above vectors were examined by performing Real-Time qPCR analysis.

### Real-Time qPCR analysis

TRIzol reagent (Invitrogen, Carlsbad, CA, USA) was used for total RNA extraction, which were subjected to BCA kit (Invitrogen, Carlsbad, CA, USA) for RNA quantification. PrimeScript™ 1st Strand cDNA Synthesis Kit (TaKaRa, Dalian, China) was used to reversely transcribe the RNA into complementary DNA (cDNA), and Real-Time qPCR was used to quantify genes expression at RNA levels by using the PrimeScript™ RT Master Mix (TaKaRa, Dalian, China) and 7500 Real-Time PCR system (Applied Biosystems, Carlsbad, CA, USA). The primer sequences were designed as follows: miR-556-5p (F, 5ʹ-GAT AGT AAT AAG AAA GAT GAG-3ʹ; R, 5ʹ-TGT TGA AGG TAG TAA TAA AAA-3ʹ), NLRP3 (F, 5ʹ-GCA CTG CTG AGG CTC TCT C-3ʹ; R, 5ʹ-GTA GAA GTG CTC AGC CCC AG-3ʹ), U6 (F, 5ʹ-GTG CTC GCT TCG GCA GCA CA-3ʹ; R, 5ʹ-AAA ATA TGG AAC GCT TCA CG-3ʹ), GAPDH (F, 5ʹ-GCT CCC TCT TTC TTT GCA GC-3; R, 5ʹ-GTT GTC ATG GAT GAC CTT GGC-3ʹ).

### Western Blot analysis

Total proteins extraction process was performed by using the RIPA lysis buffer purchased from Beyotime (Shanghai, China), and the total proteins were separated according to their molecular weight by using the 10% SDS-PAGE, which were then transferred onto the PVDF membranes (Millipore, MA, USA) and were blocked with the 5% nonfat milk. Then, the above membranes were probed with the primary antibodies against NLRP3 (1:1500, Abcam, UK), Gasdermin D (1:1500, Abcam, UK), cleaved caspase-1 (1:1500, Abcam, UK), IL-1β (1:2000, Abcam, UK), IL-18 (1:1000, Abcam, UK) and GAPDH. (1:2500, Abcam, UK) at 4 °C overnight, which were subsequently incubated with the secondary antibodies (1:3000, Abcam, UK) for 2 h at room temperature. An Enhanced Chemiluminescence (ECL, Bio-Rad, CA, USA) was used to visualize the protein bands, and the gray values were measured by the Image J software to represent protein expressions.

### MTT assay for cell proliferation

The cells were seeded into the 96-well plates at the density of 1 × 10^5^ cells per well and were subjected to high-dose cisplatin (25 μg/ml) treatment for 0 h, 6 h, 12 h, 18 h, 24 h and 48 h, and the cells were incubated with 20 μL MTT solution at concentration of 5 mg/mL for 4 h in the incubator. Then, the supernatants were carefully removed, and 150 μL dimethyl sulfoxide (DMSO) was added to the well and incubated with the cells for 10 min to dissolve the Formazan, the cells were fully vortexed and a microplate reader (ThermoFisher Scientific, USA) was used to measure the optical density (OD) values at 570 nm, which was used to represent cell viability.

### Examination of cell apoptosis

The NSCLC cells were subjected to high-dose cisplatin (25 μg/ml) exposure for 24 h, and a Apoptosis kit (Beyotime, Shanghai, China) was purchased to evaluate cell apoptosis ratio in keeping with manufacturer’s experimental protocol. Specifically, the cells were subsequently stained with Annexin V-FITC and PI for 15 min without light at room temperature, and a flow cytometer (FCM, BD Bioscience, CA, USA) was employed to measure cell apoptosis ratio.

### Dual-luciferase reporter gene system assay

The bioinformatics analysis was performed to predict the targeting sites in miR-556-5p and 3ʹUTR of NLRP3 mRNA, which were verified by using the following dual-luciferase reporter gene system assay as previously described [43]. In brief, the binding sites in wild-type NLRP3 (Wt-NLRP3) were mutated as mutant NLRP3 (Mut-NLRP3), and were cloned into the luciferase reporter vectors (Promega, WI, USA). The miR-NC, miR-556-5p mimc, and NLRP3 luciferase vectors were respectively co-transfected into the A549 and H1299 cells, and a commercial Dual-luciferase assay kit (Promega, WI, USA) was obtained to measure relative luciferase activities following the manufacturer’s instructions.

### RNA pull-down assay

The biotin-labeled NLRP3 probe containing the NLRP3-miR-556-5p binding sites were designed and constructed by Sangon Biotech (Shanghai, China), and the following RNA pull-down assay was performed to validate those sites. Specifically, the NSCLC cells were sequentially subjected to fixation, lysis and sonication, which were further centrifuged and the supernatants were collected for further analysis. Part of the supernatants were sued as input, and the NLRP3-probe-streptavidin Dynabeads (Invitrogen, USA) were used to incubate with the rest of the supernatants to facilitate the combination of NLRP3 probes and miR-556-5p, and miR-556-5p was released from the complex by incubating with lysis buffer and Proteinase K. Finally, the Real-Time qPCR analysis was performed to evaluate relative miR-556-5p enrichment.

### Evaluation of IL-1β and IL-18 cytokines secretion

The CR-NSCLC cells’ supernatants were collected, and the corresponding ELISA kits were purchased from Elabscience (Wuhan, China) was bought to measure the expression levels of the IL-1β and IL-18, and the detailed experimental procedures could be found in their instructions.

### Establishment of tumor-bearing mice models

The nine six-week-old male BALB/c nude mice were purchased and fed in the specific-pathogen-free (SPF) conditions in the Research Animal Center Affiliated to Harbin Medical University, and the A549/DDP cells with or without miR-556-5p downregulation were injected into the dorsal flank of the mice at the density of 1 × 10^7^ cells per mouse, and were subcutaneously injected with cisplatin into the tumors at the concentration of 5 mg/kg at 0 day, 7 day, 14 day, 21 day and 28 day, respectively. The mice tumor volumes were monitored, and at 28 days post-injection, the mice were anesthetized and sacrificed, and the tumors were obtained and weighed to evaluate *in vivo* tumorigenesis of the A549/DDP cells.

### Data collection and analysis

Statistical analysis in this study was performed using SPSS 18.0 software. Data was represented as mean ± standard deviation (SD), and the differences between two or multiple groups were respectively examined by using student’s t-test and one-way ANOVA. The correlation analysis was performed using Pearson’s correlation. *P value < 0.05 was considered as statistical significance.

## Results

### Upregulated miR-556-5p was relevant to cisplatin-resistance in NSCLC tissues and cells

**Figure 1.**
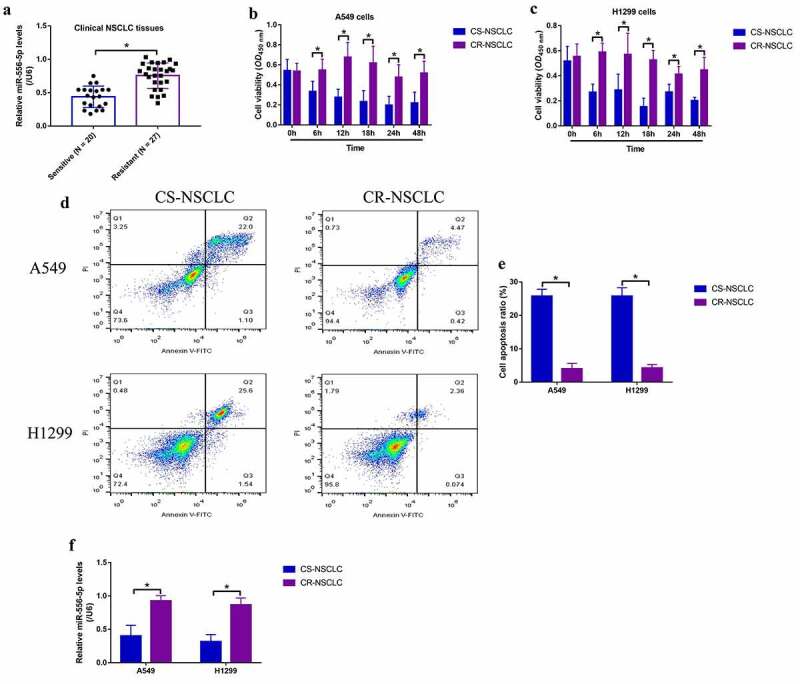
Upregulated miR-556-5p predicted cisplatin-resistance in NSCLC. (a) The cancer tissues were collected from NSCLC patients with cisplatin-sensitive and resistant properties, and miR-556-5p levels in the above tissues were examined by Real-Time qPCR analysis. (b, c) The CS-NSCLC and CR-NSCLC cells were exposed to high-dose cisplatin for 0 h, 6 h, 12 h, 18 h, 24 h and 48 h, and MTT assay was performed to evaluate cell viability. (d) The NSCLC cells were respectively stained with Annexin V-FITC and PI, and a flow cytometer was used to examine cell apoptosis ratio. (f) The expression levels of miR-556-5p in CS-NSCLC and CR-NSCLC cells were examined by Real-Time qPCR. Each experiment had 3 repetitions, and *P < 0.05

Although miR-556-5p involves in regulating cancer progression in prostate cancer [23], triple-negative breast cancer [24] and meningioma [25], its role in regulating cisplatin-resistance in NSCLC had not been investigated. To explore this issue, the NSCLC tissues collected from patients with (N = 27) and without (N = 20) cisplatin-resistant properties were examined by Real-Time qPCR, and we expectedly found that miR-556-5p was significantly enriched in the cisplatin-resistant tissues, in contrast with the corresponding cisplatin-sensitive tissues (Figure 1(a)). In addition, miR-556-5p levels were related with TNM stage in NSCLC patients, whereas its expressions had nothing to do with other clinical features, such as age, gender and smoking status (Table 1). Next, the parental cisplatin-sensitive NSCLC (CR-NSCLC) cells (A549 and H1299) were exposed to continuous low-dose cisplatin exposure to generate cisplatin-resistant NSCLC (CR-NSCLC) cells (A549 and H1299) as previously described [44], and the above cells were then subjected to high-dose cisplatin stimulation (25 μg/ml). As shown in Figure 1(b,c) the MTT assay results showed that high-dose cisplatin significantly suppressed cell viability in the CS-NSCLC cells but not in the CR-NSCLC cells. Consistently, the following FCM results verified that high-dose cisplatin especially induced cell apoptosis in the CS-NSCLC cells (Figure 1(d,e)), implying that the CR-NSCLC cells were successfully generated in this study. Then, the following Real-Time qPCR was conducted, and we found that miR-556-5p was significantly upregulated by low-dose cisplatin treatments in the CR-NSCLC cells comparing to the corresponding CS-NSCLC cells (Figure 1(f)), which were supported by the clinical results.

### Knock-down of miR-556-5p increased cisplatin-sensitivity in CR-NSCLC cells

**Figure 2.**
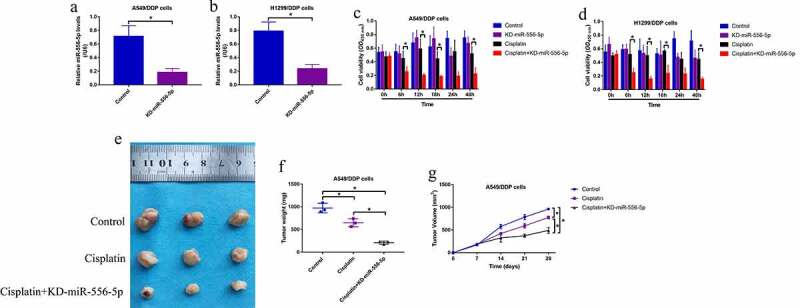
Downregulation of miR-556-5p increased cisplatin-sensitivity in CR-NSCLC cells. (a, b) The inhibitor for miR-556-5p was delivered into the A549/DDP and H1299/DDP cells for its downregulation, and the transfection efficiency was examined by Real-Time qPCR. (c, d) Cell viability in the CR-NSCLC cells were measured by performing the MTT assay. The *in vivo* xenograft tumor bearing mice models were established, and (e, f) tumor weight and (g) volume were monitored, each group had 3 mice. Each experiment had 3 repetitions, and *P < 0.05

Given that the expression levels of miR-556-5p are altered by continuous low-dose cisplatin exposure in the CR-NSCLC cells, and miR-556-5p has also been verified to be closely associated with cancer progression [23–25], it was reasonable to speculate that miR-556-5p involved in the regulation of cisplatin-resistance in NSCLC. To validate this hypothesis, the inhibitor for miR-556-5p was synthesized according to the previous studies [42,43], which were further transfected into the CR-NSCLC cells for miR-556-5p downregulation (Figure 2(a,b)). The MTT assay results in Figure 2(c,d) showed that miR-556-5p knockdown significantly inhibited cell viability in high-dose cisplatin-treated CR-NSCLC cells, while miR-556-5p inhibitor alone had little effects on cell proliferation in the CR-NSCLC cells. Consistently, cisplatin especially suppressed cell viability in miR-556-5p-deficient CR-NSCLC cells in a dose-dependent manner at 48 h post-treatment (Figure S4A, B). In addition, the A549/DDP cells with or without miR-556-5p ablation were used to establish xenograft tumor-bearing mice models, and the Real-Time qPCR assay results evidenced that miR-556-5p was successfully silenced in the mice tumor tissues (Figure S6). The following results showed that miR-556-5p knockdown aggravated the inhibiting effects of cisplatin treatment on tumor growth *in vivo* (Figure 2(e–g)), implying that targeting miR-556-5p was effective to increase cisplatin-sensitivity in NSCLC.

### Upregulation of miR-556-5p promoted cisplatin-resistance in CS-NSCLC cells

**Figure 3.**
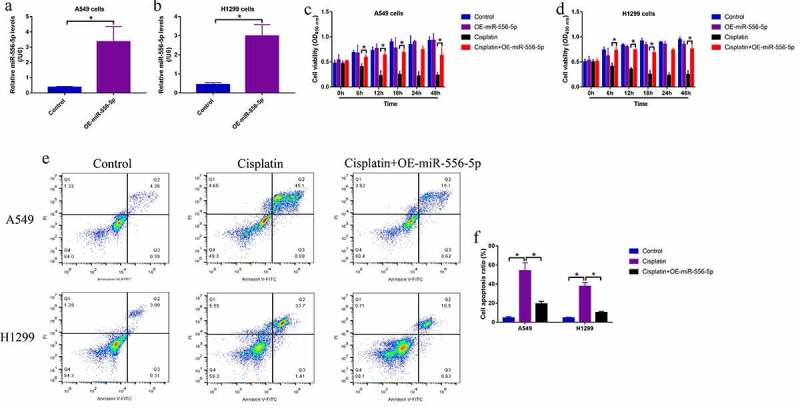
Overexpression of miR-556-5p promoted cisplatin-resistance in CS-NSCLC cells. (a, b) The miR-556-5p mimic was transfected into the A549 and H1299 cells for its upregulation. (c, d) Upregulated miR-556-5p increased cell viability in high-dose cisplatin-treated CS-NSCLC cells, as determined by MTT assay. (e, f) Cell apoptosis in A549 and H1299 cells was determined by Annexin V-FITC and PI double staining assay. Each experiment had 3 repetitions, and *P < 0.05

Since knockdown of miR-556-5p increases cisplatin-sensitivity in CR-NSCLC cells, we next explored whether upregulation of miR-556-5p also regulated cisplatin-resistance in CS-NSCLC cells. The miR-556-5p mimic were delivered into the CS-NSCLC cells for its upregulation (Figure 3(a,b)), and the cells were next subjected to high-dose cisplatin and were grouped as follows: Control, cisplatin alone group and cisplatin + miR-556-5p mimic group. The MTT assay results showed that cisplatin suppressed cell viability in CS-NSCLC cells, which were reversed by upregulating miR-556-5p (Figure 3(c,d)). Similarly, the FCM assay was conducted to measure cell apoptosis ratio, and we expectedly found that high-dose cisplatin was prone to induce cell apoptosis in the CS-NSCLC cells without miR-556-5p overexpression (Figure 3(e,f)), indicating that upregulation of miR-556-5p increased cisplatin-resistance in CS-NSCLC cells.

### MiR-556-5p ablation triggered NLRP3-mediated cell pyroptosis in CR-NSCLC cells

**Figure 4.**
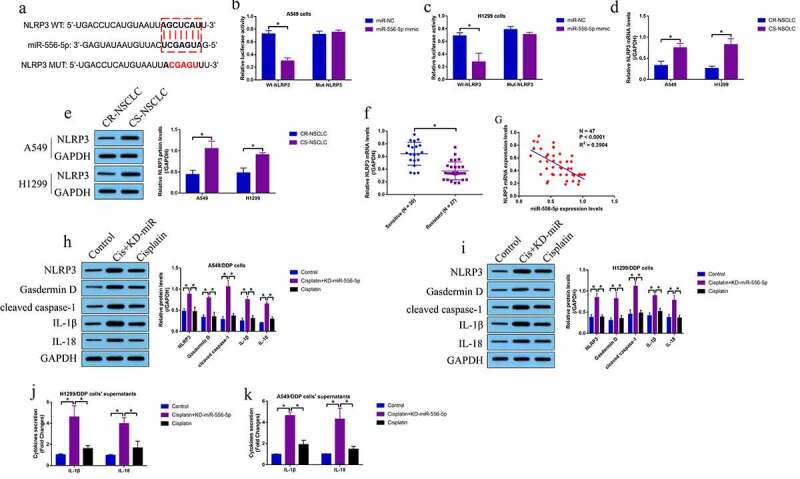
Knock-down of miR-556-5p promoted NLRP3-mediated cell pyroptosis in high-dose cisplatin treated CR-NSCLC cells. (a) The targeting sites between miR-556-5p and 3ʹUTR of NLRP3 mRNA were predicted, which were validated by performing the following (b, c) dual-luciferase reporter gene system assay. (d) The mRNA and (e) protein level of NLRP3 in NSCLC cells were determined by Real-Time qPCR and Western Blot analysis (The uncropped WB images could be found in Figure S1). (f) NLRP3 mRNA was downregulated in the cisplatin-resistant NSCLC tissues, and (g) correlations between NLRP3 mRNA and miR-556-5p in clinical tissues were analyzed by Pearson correlation analysis. (h, i) The pyroptosis associated signatures were detected by Western Blot analysis (The uncropped WB images were shown in Figure S2-S3), and (j, k) ELISA was performed to evaluate IL-1β and IL-18 secretion in the CR-NSCLC cells’ supernatants. Each experiment had 3 repetitions, and *P < 0.05

According to the existed information, miRNAs exert their biological functions by targeting the 3ʹ untranslated region (3ʹUTR) of their downstream target mRNAs [45]. Through performing the bioinformatics analysis, we evidenced that the 3ʹ UTR of NLRP3 mRNA could be targeted by miR-556-5p (Figure 4(a)), which were validated by performing the following dual-luciferase reporter gene system assay (Figure 4(b,c)) and RNA pull-down assay (Figure S5A, B). Then, the Real-Time qPCR and Western Blot analysis were performed, and the results showed that NLRP3 was significantly downregulated in the CR-NSCLC cells, in contrast with the CS-NSCLC cells (Figure 4(d,e)), which were supported by the clinical results that NLRP3 tended to be downregulated in the CR-NSCLC tissues (Figure 4(f)), and the expression levels of miR-556-5p and NLRP3 mRNA showed negative correlations in the NSCLC tissues (Figure 4(g)). In addition, according to the previous literatures [33–35], NLRP3-mediated cell pyroptosis is closely associated with cisplatin-sensitivity, and this study evidenced that knockdown of miR-556-5p upregulated NLRP3, Gasdermin D, cleaved Caspase-1, IL-1β and IL-18 to trigger pyroptotic cell death in high-dose cisplatin treated CR-NSCLC cells (Figure 4(h,i)). Consistently, miR-556-5p ablation promoted IL-1β and IL-18 secretion in the supernatants of the CR-NSCLC cells, as determined by ELISA (Figure 4(j,k)). Moreover, upregulation of miR-556-5p suppressed NLRP3 mRNA levels in the cisplatin-treated CS-NSCLC cells (Figure S5C).

### Silencing of miR-556-5p promoted CR-NSCLC cell death through activating NLRP3-mediated pyroptotic cell death

**Figure 5.**
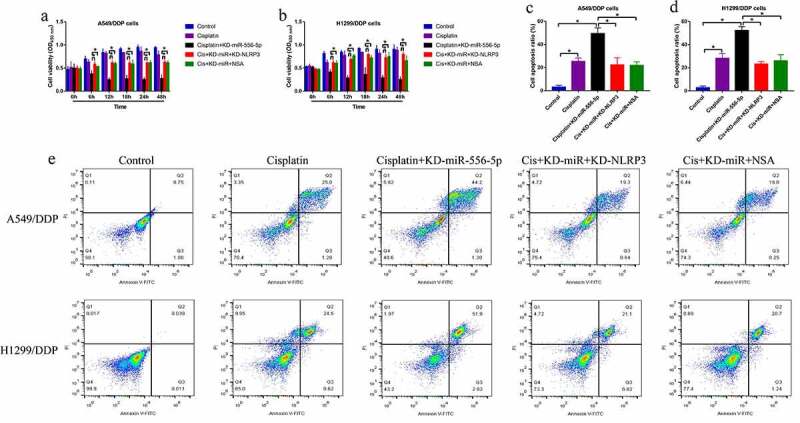
Silence of miR-556-5p increased cisplatin-sensitivity in CR-NSCLC cells via inducing NLRP3-mediated pyroptotic cell death. (a, b) Cell viability was determined by MTT assay, (c–e) and flow cytometer was employed to measure cell apoptosis ratio in A549/DDP and H1299/DDP cells. Each experiment had 3 repetitions, and *P < 0.05

Given that miR-556-5p is capable of regulating both cisplatin-sensitivity and NLRP3-mediated cell pyroptosis, and activation of pytoptotic cell death increases cisplatin-sensitivity in NSCLC, we conjectured that silence of miR-556-5p might increase cisplatin-sensitivity in NSCLC via recovering NLRP3-mediated cell pyroptosis. To validate the hypothesis, the CR-NSCLC cells were subjected to miR-556-5p knockdown (KD-miR-556-5p), NLRP3 downregulation (KD-NLRP3) and pyroptosis inhibitor NSA, and the cells were divided into five groups as follows: Control, cisplatin alone, cisplatin + KD-miR-556-5p, cisplatin + KD-miR-556-5p + KD-NLRP3, and cisplatin + KD-miR-556-5p + NSA. As shown in Figure 5(a,b), both NLRP3 downregulation and NSA abrogated the inhibiting effects of miR-556-5p downregulation on cell viability in CR-NSCLC cells. The above results were supported by the FCM results that knock-down of miR-556-5p aggravated cisplatin-induced cell apoptosis in CR-NSCLC cells, which were partially reversed by silencing NLRP3 and NSA co-treatment (Figure 5(c–e)), indicating that miR-556-5p ablation increased cisplatin-sensitivity in NSCLC cells via activating NLRP3-mediated cell pyroptosis.

## Discussion

Cisplatin-resistance is a huge obstacle to make this drug ineffective to treat NSCLC in clinic [13–15], and uncovering the potential underlying mechanisms will help to solve this problem. According to recent publications [16,44,46,47], miRNAs with post-transcriptional activities are crucial for regulating cancer progression and drug resistance, and multiple miRNAs that regulate cisplatin-resistance in NSCLC have been screened out, such as let-7 miRNA [16], miR-497-5p [46], miR-130a [47] and miR-377-3p [44]. Among all the miRNAs, miR-556-5p has also been reported to be closely associated with cancer progression, and its role in regulating cancer development varies according to differential cancer types [23–25]. However, up until now, it is still unclear whether miR-556-5p regulated drug resistance during chemotherapy. Thus, the present study was designed, and we found that miR-556-5p tended to ben enriched in CR-NSCLC tissues and cells. Further experiments validated that knock-down of miR-556-5p enhanced the cytotoxic effects of cisplatin treatment on CR-NSCLC cells, and conversely, upregulation of miR-556-5p promoted cisplatin-resistance in CS-NSCLC cells, which were partially supported by the previous data in other types of cancers [23–25], indicating that silencing of miR-556-5p increased cisplatin-sensitivity in NSCLC.

MiRNAs are a group of small non-coding RNAs that regulate cellular functions through targeting the 3ʹUTR of their downstream target genes, resulting in their degradation and inhibition [45], and previous studies suggest that miR-556-5p is capable of suppressing multiple genes expression, such as TCF12 [25], YAP1 [24] and PPP2R2A [23]. Interestingly, miR-556-5p is reported to regulate NLRP3-mediated cell pyroptosis in OVA-induced AR mice models [43], suggesting that NLRP3 is the downstream target of miR-556-5p, which were supported by our experiments that miR-556-5p targeted the 3ʹUTR of NLRP3 mRNA for its inhibition in NSCLC, and the expression levels of miR-556-5p and NLRP3 mRNA showed negative correlations in the clinical NSCLC tissues. According to the existed information that NLRP3-mediated pyroptotic cell death is critical for modulating cisplatin-resistance [33–35], we validated that long-term low-dose cisplatin exposure downregulated NLRP3 expressions in CR-NSCLC cells, indicating that NLRP3-mediated pyroptotic cell death was inactivated in CR-NSCLC cells compared to the corresponding CS-NSCLC cells.

Given that NLRP3 could be negatively regulated by miR-556-5p [43], we next verified that knockdown of miR-556-5p increased cisplatin-sensitivity by re-activating NLRP3-mediated cell pyroptosis in CR-NSCLC cells. Specifically, knockdown of miR-556-5p increased the expression levels of cell pyroptosis signatures in cisplatin-treated CR-NSCLC cells, suggesting the NLRP3-mediated pyroptotic cell death was re-activated by downregulating miR-556-5p. Moreover, as previously described, various types of cell death, including cell apoptosis [26–28] and pyroptosis [33–35], involve in regulating cisplatin-resistance in NSCLC, and previous literatures evidence that both apoptotic [25,42] and pyroptotic [43] cell death can be modulated by miR-556-5p, which were supported by our data that miR-556-5p downregulation promoted both cell apoptosis and pyroptosis in cisplatin-treated CR-NSCLC cells. In addition, there exist interplay between cell apoptosis and pyroptosis [36,37], and our data showed that blockage of pyroptotic cell death by NLRP3 ablation or pyroptosis inhibitor NSA abrogated the promoting effects of miR-556-5p downregulation on cell apoptosis in cisplatin treated CR-NSCLC cells, which were partially supported by the previous work [36,37], indicating that targeting miR-556-5p aggravated cieplatin-induced CR-NSCLC cell apoptosis via activating NLRP3-mediated cell pyroptosis.

## Conclusions

We concluded that silencing of miR-556-5p re-activated cisplatin-induced pyroptotic cell death via degrading NLRP3, resulting in the improvement of cisplatin-sensitivity in CR-NSCLC. This study, for the first time, investigated the role of miR-556-5p/NLRP3 axis in regulating cisplatin-resistance in NSCLC, which broadened our knowledge in this field and provide alternative treatment strategies for NSCLC treatment in clinic.

## Supplementary Material

Supplemental Material

## Data Availability

All the raw data could be obtained from the corresponding author, and the associated data had been included in the manuscript.

## Animal experiments statements

The animal experiments were conducted according to the guidelines approved by the Ethics Committee of Harbin Medical University Cancer Hospital, and the animal experiments were performed in compliance with the ARRIVE guideline.

## References

[1] Broderick SR. Adjuvant and neoadjuvant immunotherapy in non-small cell lung cancer. Thorac Surg Clin. 2020;30(2):215–220. 32327180 10.1016/j.thorsurg.2020.01.001

[2] Imyanitov EN, Iyevleva AG, Levchenko EV. Molecular testing and targeted therapy for non-small cell lung cancer: current status and perspectives. Crit Rev Oncol Hematol. 2021;157:103194. 33316418 10.1016/j.critrevonc.2020.103194

[3] Duma N, Santana-Davila R, Molina JR. Non-small cell lung cancer: epidemiology, screening, diagnosis, and treatment. Mayo Clin Proc. 2019;94(8):1623–1640. 31378236 10.1016/j.mayocp.2019.01.013

[4] Patel SA, Weiss J. Advances in the treatment of non-small cell lung cancer: immunotherapy. Clin Chest Med. 2020;41(2):237–247. 32402359 10.1016/j.ccm.2020.02.010

[5] Chiu YH, Hsu SH, Hsu HW, et al. Human non‑small cell lung cancer cells can be sensitized to camptothecin by modulating autophagy. Int J Oncol. 2018;53(5):1967–1979. 30106130 10.3892/ijo.2018.4523 PMC6192723

[6] Xing Y, Liu Y, Liu T, et al. TNFAIP8 promotes the proliferation and cisplatin chemoresistance of non-small cell lung cancer through MDM2/p53 pathway. Cell Commun Signal. 2018;16(1):43. 30064446 10.1186/s12964-018-0254-x PMC6069800

[7] Jeong Y, Hellyer JA, Stehr H, et al. Role of KEAP1/NFE2L2 mutations in the chemotherapeutic response of patients with non-small cell lung cancer. Clin Cancer Res. 2020;26(1):274–281. 31548347 10.1158/1078-0432.CCR-19-1237 PMC6942632

[8] Xu P, Jiang L, Yang Y, et al. PAQR4 promotes chemoresistance in non-small cell lung cancer through inhibiting Nrf2 protein degradation. Theranostics. 2020;10(8):3767–3778. 32206121 10.7150/thno.43142 PMC7069097

[9] Ma Z, Cai H, Zhang Y, et al. MiR-129-5p inhibits non-small cell lung cancer cell stemness and chemoresistance through targeting DLK1. Biochem Biophys Res Commun. 2017;490(2):309–316. 28619508 10.1016/j.bbrc.2017.06.041

[10] Yu S, Ao Z, Wu Y, et al. ZNF300 promotes chemoresistance and aggressive behaviour in non-small-cell lung cancer. Cell Prolif. 2020;53(11):e12924. 33078469 10.1111/cpr.12924 PMC7653252

[11] Tang D, Zhao D, Wu Y, et al. The miR-3127-5p/p-STAT3 axis up-regulates PD-L1 inducing chemoresistance in non-small-cell lung cancer. J Cell Mol Med. 2018;22(8):3847–3856. 29726585 10.1111/jcmm.13657 PMC6050495

[12] Ichikawa A, Fujita Y, Hosaka Y, et al. Chaperone-mediated autophagy receptor modulates tumor growth and chemoresistance in non-small cell lung cancer. Cancer Sci. 2020;111(11):4154–4165. 32860290 10.1111/cas.14629 PMC7648026

[13] Hu C, Zhang M, Moses N, et al. The USP10-HDAC6 axis confers cisplatin resistance in non-small cell lung cancer lacking wild-type p53. Cell Death Dis. 2020;11(5):328. 32382008 10.1038/s41419-020-2519-8 PMC7206099

[14] Wang W, Zhao M, Cui L, et al. Characterization of a novel HDAC/RXR/HtrA1 signaling axis as a novel target to overcome cisplatin resistance in human non-small cell lung cancer. Mol Cancer. 2020;19(1):134. 32878625 10.1186/s12943-020-01256-9 PMC7466461

[15] Wu H, Mu X, Liu L, et al. Bone marrow mesenchymal stem cells-derived exosomal microRNA-193a reduces cisplatin resistance of non-small cell lung cancer cells via targeting LRRC1. Cell Death Dis. 2020;11(9):801. 32978367 10.1038/s41419-020-02962-4 PMC7519084

[16] Hong W, Xue M, Jiang J, et al. Circular RNA circ-CPA4/ let-7 miRNA/PD-L1 axis regulates cell growth, stemness, drug resistance and immune evasion in non-small cell lung cancer (NSCLC). J Exp Clin Cancer Res. 2020;39(1):149. 32746878 10.1186/s13046-020-01648-1 PMC7397626

[17] Zamagni A, Pasini A, Pirini F, et al. CDKN1A upregulation and cisplatin‑pemetrexed resistance in non‑small cell lung cancer cells. Int J Oncol. 2020;56(6):1574–1584. 32236605 10.3892/ijo.2020.5024 PMC7170038

[18] Chen L, Zhu Q, Lu L, et al. MiR-132 inhibits migration and invasion and increases chemosensitivity of cisplatin-resistant oral squamous cell carcinoma cells via targeting TGF-β1. Bioengineered. 2020;11(1):91–102. 31906769 10.1080/21655979.2019.1710925 PMC6961592

[19] Liu X, Zhou X, Chen Y, et al. miR-186-5p targeting SIX1 inhibits cisplatin resistance in non-small-cell lung cancer cells (NSCLCs). Neoplasma. 2020;67(1):147–157. 31686523 10.4149/neo_2019_190511N420

[20] Yang W, Xiao W, Cai Z, et al. miR-1269b drives cisplatin resistance of human non-small cell lung cancer via modulating the PTEN/PI3K/AKT signaling pathway. Onco Targets Ther. 2020;13:109–118. 32021259 10.2147/OTT.S225010 PMC6954839

[21] Sun B, Hu N, Cong D, et al. MicroRNA-25-3p promotes cisplatin resistance in non-small-cell lung carcinoma (NSCLC) through adjusting PTEN/PI3K/AKT route. Bioengineered. 2021;12(1):3219–3228. 34266345 10.1080/21655979.2021.1939577 PMC8806525

[22] Wei L, Jiang J. Targeting the miR-6734-3p/ZEB2 axis hampers development of non-small cell lung cancer (NSCLC) and increases susceptibility of cancer cells to cisplatin treatment. Bioengineered. 2021;12(1):2499–2510. 34107856 10.1080/21655979.2021.1936891 PMC8806905

[23] Zhao W, Cao L, Zeng S, et al. Upregulation of miR-556-5p promoted prostate cancer cell proliferation by suppressing PPP2R2A expression. Biomed Pharmacother. 2015;75:142–147. 26297546 10.1016/j.biopha.2015.07.015

[24] Zhou Y, Liu X, Lan J, et al. Circular RNA circRPPH1 promotes triple-negative breast cancer progression via the miR-556-5p/YAP1 axis. Am J Transl Res. 2020;12(10):6220–6234. 33194025 PMC7653573

[25] Zhang Y, Yu R, Li Q, et al. SNHG1/miR-556-5p/TCF12 feedback loop enhances the tumorigenesis of meningioma through Wnt signaling pathway. J Cell Biochem. 2020;121(2):1880–1889. 31692066 10.1002/jcb.29423

[26] Moro M, Caiola E, Ganzinelli M, et al. Metformin enhances cisplatin-induced apoptosis and prevents resistance to cisplatin in co-mutated KRAS/LKB1 NSCLC. J Thorac Oncol. 2018;13(11):1692–1704. 30149143 10.1016/j.jtho.2018.07.102

[27] Cruz-Bermúdez A, Laza-Briviesca R, Vicente-Blanco RJ, et al. Cisplatin resistance involves a metabolic reprogramming through ROS and PGC-1α in NSCLC which can be overcome by OXPHOS inhibition. Free Radic Biol Med. 2019;135:167–181. 30880247 10.1016/j.freeradbiomed.2019.03.009

[28] Ma Y, Yuwen D, Chen J, et al. Exosomal transfer of cisplatin-induced miR-425-3p confers cisplatin resistance in NSCLC through activating autophagy. Int J Nanomedicine. 2019;14:8121–8132. 31632022 10.2147/IJN.S221383 PMC6790351

[29] Wang Y, Hao F, Nan Y, et al. PKM2 inhibitor shikonin overcomes the cisplatin resistance in bladder cancer by inducing necroptosis. Int J Biol Sci. 2018;14(13):1883–1891. 30443191 10.7150/ijbs.27854 PMC6231221

[30] Liu L, Fan J, Ai G, et al. Berberine in combination with cisplatin induces necroptosis and apoptosis in ovarian cancer cells. Biol Res. 2019;52(1):37. 31319879 10.1186/s40659-019-0243-6 PMC6637630

[31] Zhang H, Deng T, Liu R, et al. CAF secreted miR-522 suppresses ferroptosis and promotes acquired chemo-resistance in gastric cancer. Mol Cancer. 2020;19(1):43. 32106859 10.1186/s12943-020-01168-8 PMC7045485

[32] Zhang X, Sui S, Wang L, et al. Inhibition of tumor propellant glutathione peroxidase 4 induces ferroptosis in cancer cells and enhances anticancer effect of cisplatin. J Cell Physiol. 2020;235(4):3425–3437. 31556117 10.1002/jcp.29232 PMC13482695

[33] Peng Z, Wang P, Song W, et al. GSDME enhances Cisplatin sensitivity to regress non-small cell lung carcinoma by mediating pyroptosis to trigger antitumor immunocyte infiltration. Signal Transduct Target Ther. 2020;5(1):159. 32839451 10.1038/s41392-020-00274-9 PMC7445264

[34] Xu X, Zhou X, Chen Z, et al. Silencing of lncRNA XIST inhibits non-small cell lung cancer growth and promotes chemosensitivity to cisplatin. Aging (Albany NY). 2020;12(6):4711–4726. 32209729 10.18632/aging.102673 PMC7138551

[35] Long K, Gu L, Li L, et al. Small-molecule inhibition of APE1 induces apoptosis, pyroptosis, and necroptosis in non-small cell lung cancer. Cell Death Dis. 2021;12(6):503. 34006852 10.1038/s41419-021-03804-7 PMC8131371

[36] He B, Shi Y, Liang Y, et al. Single-walled carbon-nanohorns improve biocompatibility over nanotubes by triggering less protein-initiated pyroptosis and apoptosis in macrophages. Nat Commun. 2018;9(1):2393. 29921862 10.1038/s41467-018-04700-z PMC6008334

[37] Pi S, Nie G, Wei Z, et al. Inhibition of ROS/NLRP3/Caspase-1 mediated pyroptosis alleviates excess molybdenum-induced apoptosis in duck renal tubular epithelial cells. Ecotoxicol Environ Saf. 2021;208:111528. 33157513 10.1016/j.ecoenv.2020.111528

[38] Shi T, Fujita K, Gong J, et al. Aspirin inhibits hepatocellular carcinoma cell proliferation in vitro and in vivo via inducing cell cycle arrest and apoptosis. Oncol Rep. 2020;44(2):457–468. 32627038 10.3892/or.2020.7630 PMC7336451

[39] Wen Z, Mai Z, Zhu X, et al. Mesenchymal stem cell-derived exosomes ameliorate cardiomyocyte apoptosis in hypoxic conditions through microRNA144 by targeting the PTEN/AKT pathway. Stem Cell Res Ther. 2020;11(1):36. 31973741 10.1186/s13287-020-1563-8 PMC6979357

[40] Zhang Y, Liu X, Bai X, et al. Melatonin prevents endothelial cell pyroptosis via regulation of long noncoding RNA MEG3/miR-223/NLRP3 axis. J Pineal Res. 2018;64(2):e12449. 10.1111/jpi.12449 29024030

[41] Mao Q, Liang XL, Zhang CL, et al. LncRNA KLF3-AS1 in human mesenchymal stem cell-derived exosomes ameliorates pyroptosis of cardiomyocytes and myocardial infarction through miR-138-5p/Sirt1 axis. Stem Cell Res Ther. 2019;10(1):393. 31847890 10.1186/s13287-019-1522-4 PMC6918658

[42] Xu Y, Gao P, Wang Z, et al. Circ-LAMP1 contributes to the growth and metastasis of cholangiocarcinoma via miR-556-5p and miR-567 mediated YY1 activation. J Cell Mol Med. 2021;25(7):3226–3238. 33675150 10.1111/jcmm.16392 PMC8034453

[43] Yu X, Wang M, Zhao H, et al. Targeting a novel hsa_circ_0000520/miR-556-5p/NLRP3 pathway-mediated cell pyroptosis and inflammation attenuates ovalbumin (OVA)-induced allergic rhinitis (AR) in mice models. Inflamm Res. 2021;70(6):719–729. 10.1007/s00011-021-01472-z 34028600

[44] Zhu X, Han J, Lan H, et al. A novel circular RNA hsa_circRNA_103809/miR-377-3p/GOT1 pathway regulates cisplatin-resistance in non-small cell lung cancer (NSCLC). BMC Cancer. 2020;20(1):1190. 33276753 10.1186/s12885-020-07680-w PMC7716498

[45] Zhao H, Wang S, Guo L, et al. Fixed differences in the 3ʹUTR of buffalo PRNP gene provide binding sites for miRNAs post-transcriptional regulation. Oncotarget. 2017;8(28):46006–46019. 28545018 10.18632/oncotarget.17545 PMC5542244

[46] Hou Z, Wang Y, Xia N, et al. Pseudogene KRT17P3 drives cisplatin resistance of human NSCLC cells by modulating miR-497-5p/mTOR. Cancer Sci. 2021;112(1):275–286. 33179318 10.1111/cas.14733 PMC7780050

[47] Zhang T, Zhang P, Li HX. CAFs-derived exosomal miRNA-130a confers cisplatin resistance of NSCLC cells through PUM2-dependent packaging. Int J Nanomedicine. 2021;16:561–577. 33542625 10.2147/IJN.S271976 PMC7851405

