# Supplementary material for: Knock-down of microRNA miR-556-5p increases cisplatin-sensitivity in non-small cell lung cancer (NSCLC) via activating NLR family pyrin domain containing 3 (NLRP3)-mediated pyroptotic cell death
Source: Bioengineered. 2021 Sep 7;12(1):6332–42. doi: 10.1080/21655979.2021.1971502 (PMC8806686; doi:10.1080/21655979.2021.1971502)
Supplement: Supplemental Material [file KBIE_A_1971502_SM8610.docx]

**Supplementary figures and figure legends**


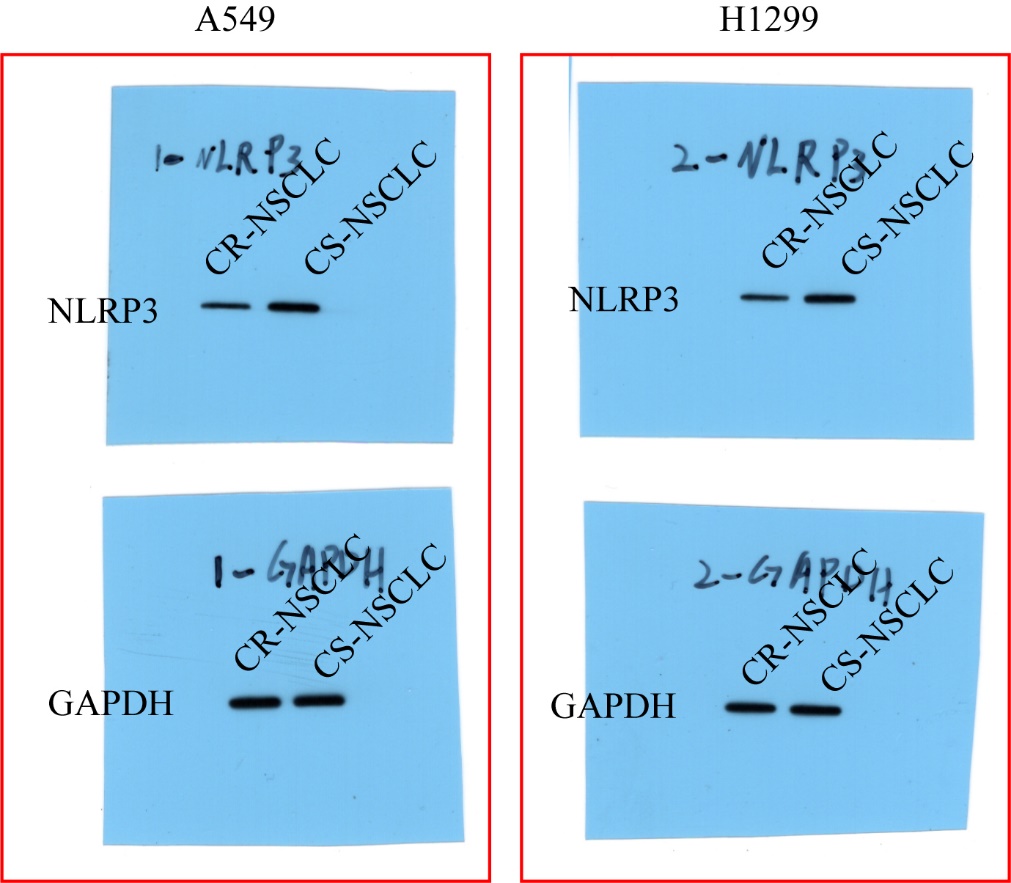


Figure S1. The uncropped WB images for Figure 4E.


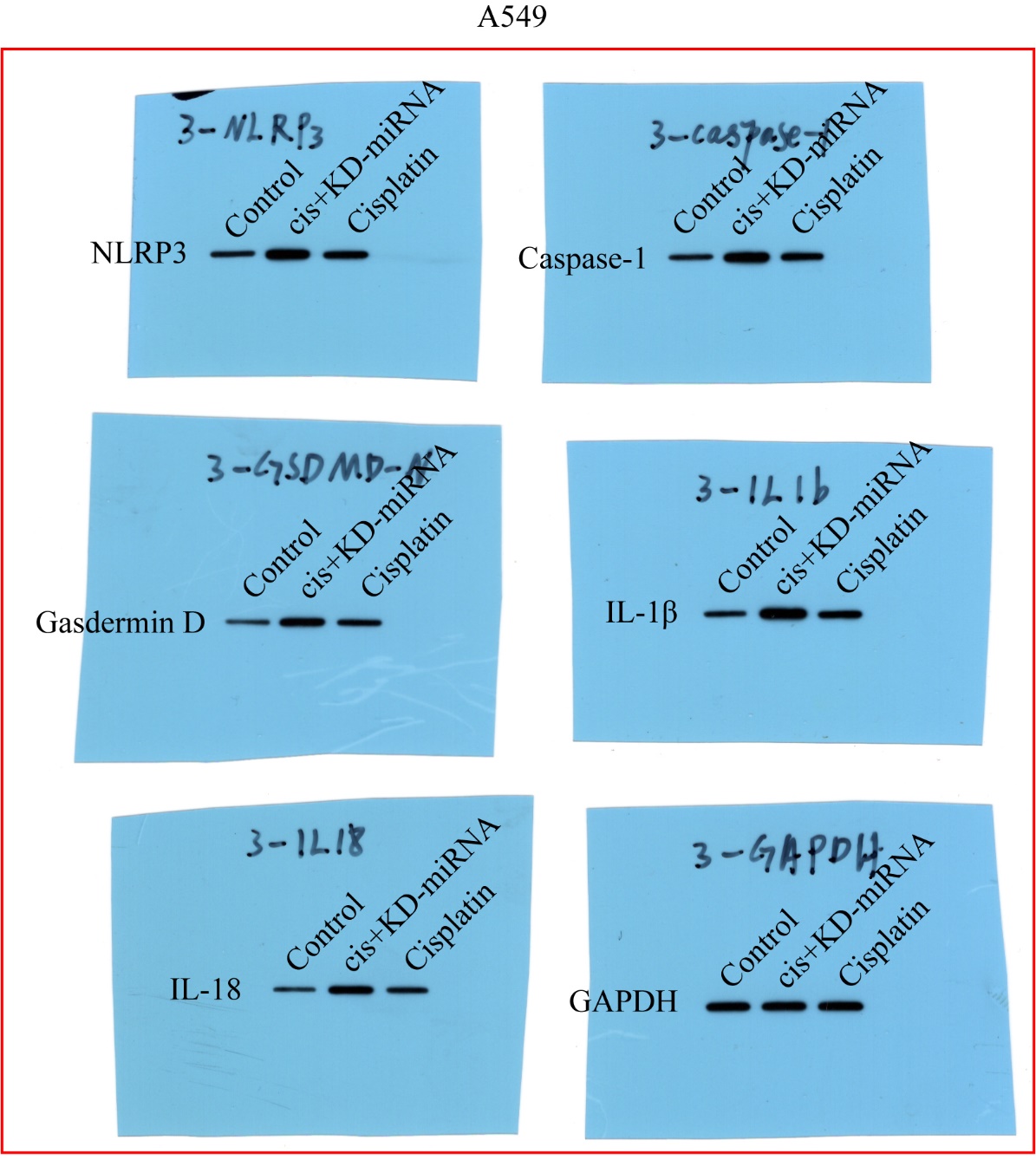


Figure S2. The uncropped WB images for Figure 4H.


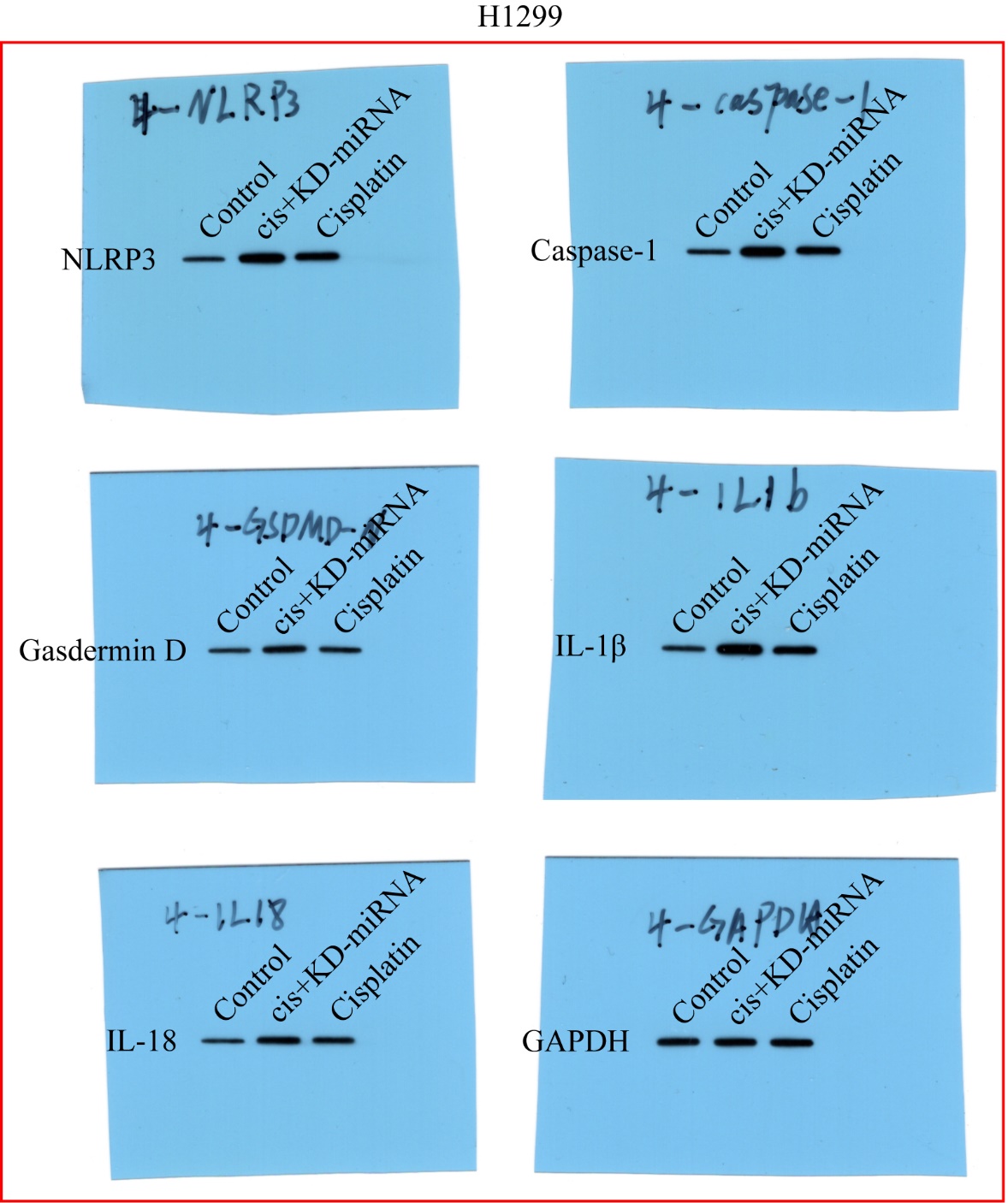


Figure S3. The uncropped WB images for Figure 4I.


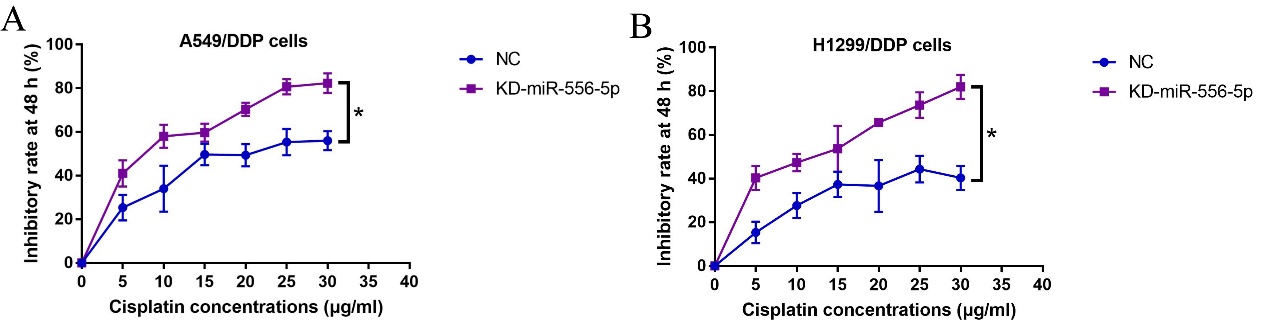


Figure S4. The inhibiting rate of different doses of cisplatin on CR-NSCLC cells at 48h post-treatment, as determined by MTT assay. **P* < 0.05.


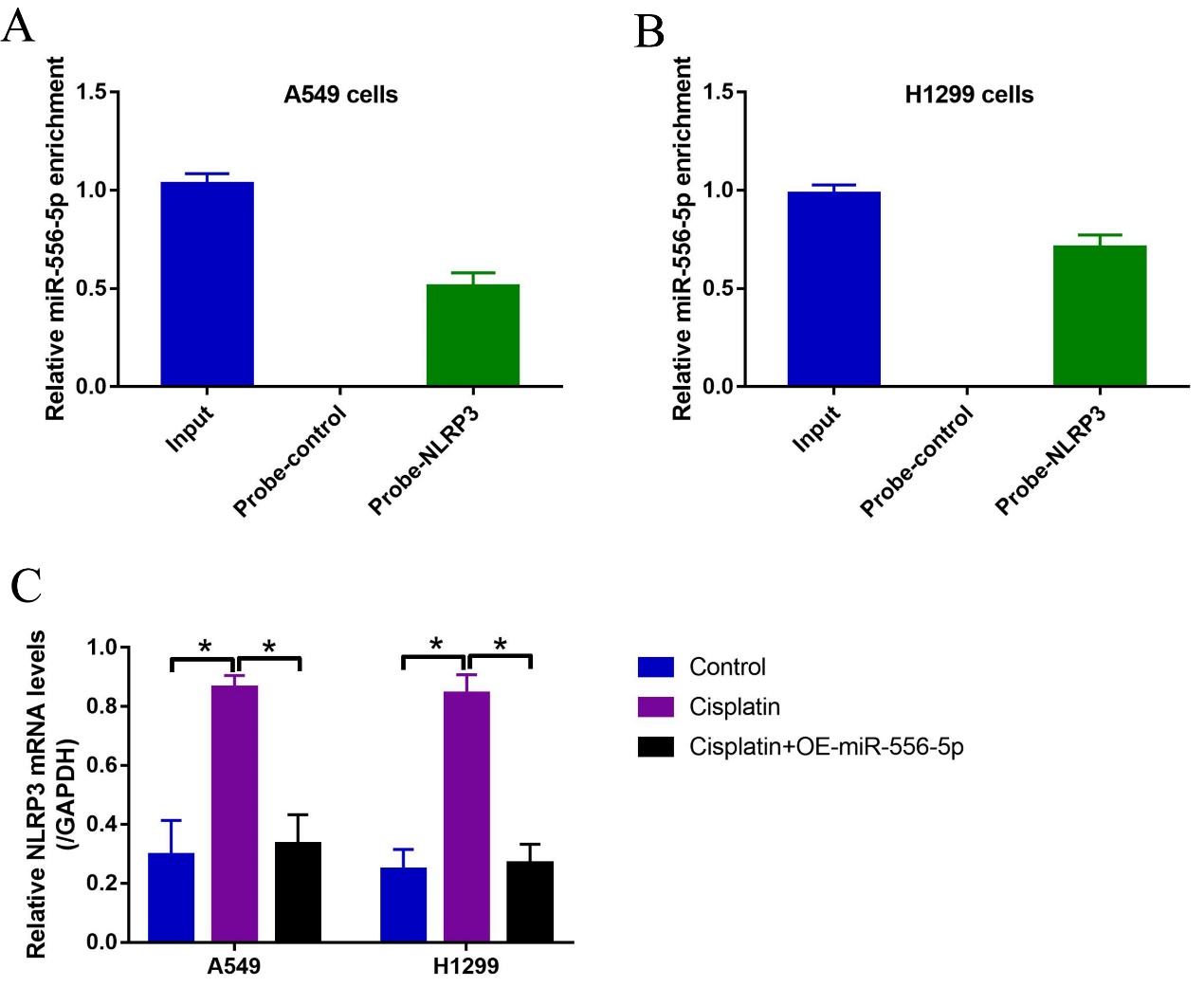


Figure S5. (A, B) RNA pull-down assay was performed to validate the targeting sites between miR-556-5p and NLRP3 mRNA. (C) The regulating effects of miR-556-5p overexpression on NLRP3 mRNA levels in the cisplatin-treated CS-NSCLC cells were determined by Real-Time qPCR. **P* < 0.05.


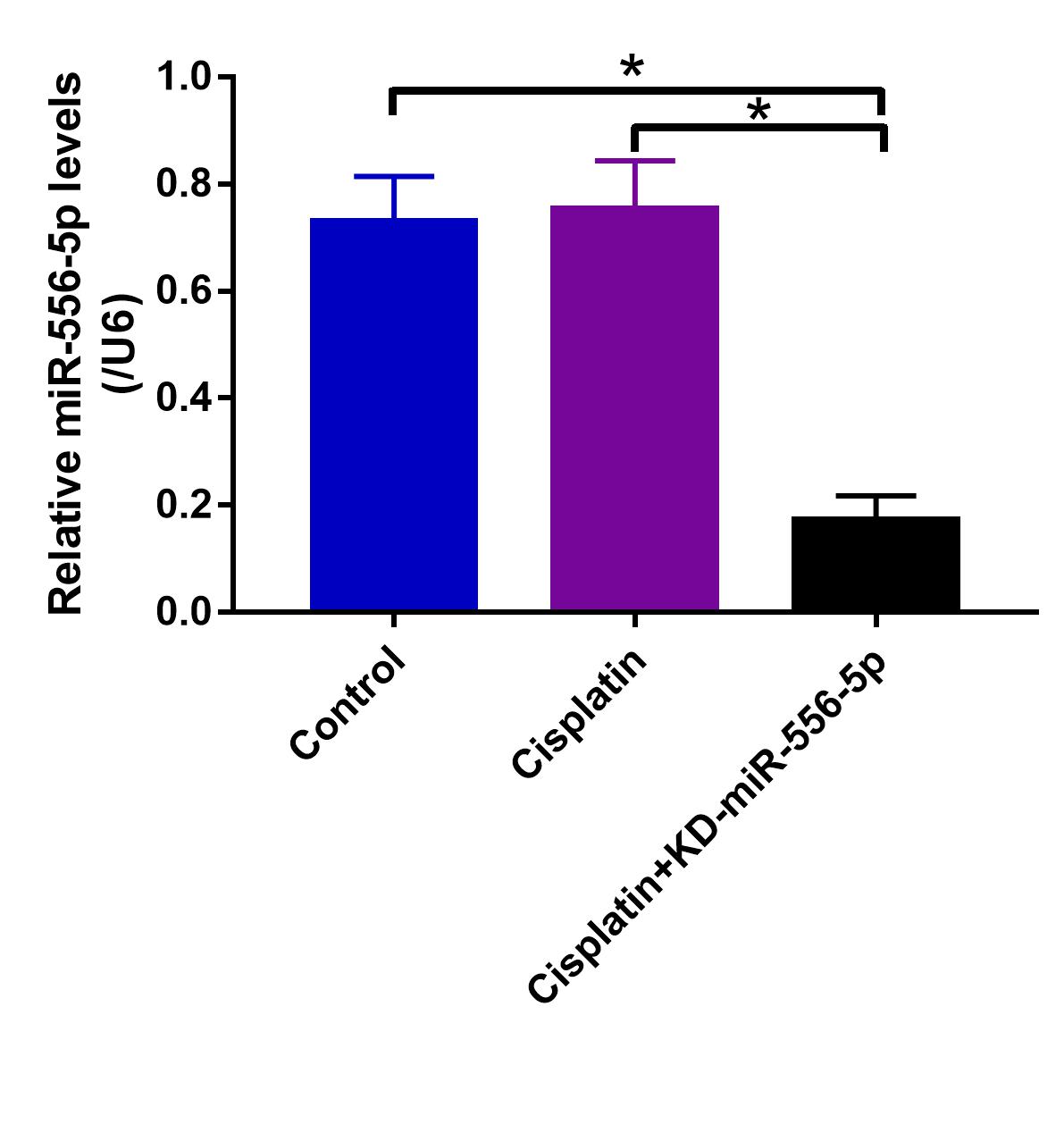


Figure S6. The expression levels of miR-556-5p in mice tumor tissues were determined by performing Real-Time qPCR analysis. **P* < 0.05.
